# Clinicopathologic Analysis of Five Patients with *POLE*-Mutated Colorectal Cancer in a Single Korean Institute

**DOI:** 10.3390/diagnostics15080972

**Published:** 2025-04-11

**Authors:** Harim Oh, Inho Jang, Jinha Hwang, Soohyeon Lee, Jungsuk An, Jongmin Sim

**Affiliations:** 1 Department of Pathology, Korea University Anam Hospital, Korea University College of Medicine, 73 Anam-ro, Seongbuk-gu, Seoul 02841, Republic of Korea; harim1028@gmail.com; 2 Department of Medicine, Korea University College of Medicine, Seoul 02841, Republic of Korea; yhbestbest@korea.ac.kr; 3 Department of Laboratory Medicine, Korea University Anam Hospital, Korea University College of Medicine, Seoul 02841, Republic of Korea; jinha1226@gmail.com; 4 Division of Medical Oncology, Department of Internal Medicine, Korea University Anam Hospital, Korea University College of Medicine, Seoul 02841, Republic of Korea; soohyeon_lee@korea.ac.kr; 5 Department of Pathology, Ewha Womans University Mokdong Hospital, Ewha Womans University College of Medicine, Seoul 07985, Republic of Korea; anbox@naver.com

**Keywords:** colon cancer, rectal cancer, *POLE* mutation

## Abstract

**Background/Objectives**: Mutations in RAS/RAF are common in colorectal cancer (CRC) and play a pivotal role in guiding treatment selection. With the recent advent of immunotherapy, microsatellite (MSI) status, tumor mutation burden (TMB), and *POLE* mutations, particularly those leading to high TMB, have gained importance in CRC. This study aimed to examine the clinicopathological characteristics of patients with CRC with *POLE* mutations. **Methods**: We identified *POLE* mutations in patients with colorectal cancer who had available next-generation sequencing (NGS) results from a single institute in Korea. RAS/RAF status, MSI status, and TMB were evaluated, and based on the TMB results, patients with *POLE* mutations were classified as having either pathogenic or non-pathogenic mutations. After excluding non-Korean patients, we compared the groups based on the presence of pathogenic *POLE* mutations. **Results**: Five *POLE* mutations (A456P, P286R, R1111W, R609W, and V922I) were identified. Only A456P and P286R were associated with an exceptionally high TMB, resulting in two patients (1.1%) being categorized as having pathogenic *POLE*. The *POLE*-mutant group showed an extremely high TMB and tended to include younger patients. Among the two pathogenic cases, one showed poor histological differentiation, and the tumors were split between the right and left colons (one in each). **Conclusions**: CRC with *POLE* mutations tend to exhibit TMB-high, occur in younger patients, localize to the right colon, and display poor histological differentiation. Given that *POLE* mutations can serve as indicators for immunotherapy, recognizing these mutations is of clinical importance.

## 1. Introduction

DNA polymerase ε, encoded by the *POLE* gene, is a critical protein that performs both polymerase and exonuclease functions during DNA synthesis [1]. Simultaneously with the synthesis of the leading strand of DNA, the proofreading activities of its exonuclease region allow for the recognition and excision of mismatched bases, thereby increasing the fidelity of new DNA replication [2]. Pathogenic mutations in the exonuclease domain result in proofreading defects, leading to the accumulation of somatic missense mutations and development of an ultra-hypermutated tumor phenotype [3,4].

DNA polymerase proofreading and DNA mismatch repair (MMR) are two critical mechanisms that ensure the high fidelity of DNA replication. Deficiency in MMR (dMMR) disrupts genomic stability, resulting in a high tumor mutational burden (TMB), accumulation of immunogenic neoantigens, and infiltration of cytotoxic T cells into the tumor microenvironment [5]. Immune checkpoint inhibitors (ICIs) have been established as the gold standard treatment for patients with dMMR colorectal cancers (CRC) [6]. Similarly, *POLE*-mutated CRCs, characterized by a hypermutated phenotype and high immunogenicity, are considered ideal candidates for immunotherapy [7]. Notably, metastatic CRC with *POLE* mutations also responds well to ICI therapy, with an overall response rate of 89% [8]. Despite this promising therapeutic potential, pathogenic *POLE* mutations are found in only 1–2% of CRC cases, and their detection requires advanced molecular techniques such as polymerase chain reaction (PCR), Sanger sequencing, and next-generation sequencing (NGS) [9,10]. Consequently, *POLE* mutations are often underdiagnosed in clinical practice, and their rarity hinders a comprehensive understanding of their clinical significance.

This study aims to comparatively review the clinical, pathologic, and molecular features of CRC with *POLE* mutation in a single South Korean institute.

## 2. Materials and Methods

### 2.1. Patients, Inclusion, and Exclusion Criteria

This study included patients diagnosed with colorectal cancer at Korea University, Anam Hospital, from May 2022 to October 2024. Patients who underwent NGS were included in this study. Clinical information was retrieved from electronic medical records, and pathological factors were evaluated by reviewing pathology reports. TNM staging was performed using the 8th American Joint Committee on Cancer (AJCC) staging system [11]. Patients diagnosed with histopathologically confirmed colorectal cancer and whose histopathology and immunohistochemistry reports were available in the electronic database were included in this study. Patients with neuroendocrine tumors were excluded to focus on the clinicopathological characteristics of CRC.

### 2.2. Molecular Analysis

The following techniques were used for the molecular and genetic analyses of patients with CRC. Targeted sequencing was performed using a TruSight Oncology 500 panel (TSO500) (Illumina, San Diego, CA, USA). The TSO500 panel v1.0 included whole exomes of 523 cancer-related genes (Supplemental Table S1). The panel used the hybrid capture method that incorporates both DNA and RNA. The sequencing method was validated on hybridization-captured adaptor ligation-based libraries using DNA extracted from ten formalin-fixed paraffin-embedded sections cut at 5 mm. Sequenced reads from the FASTQ files were aligned with the human genome assembly (hg19) using a Burrows–Wheeler aligner. The initially aligned BAM files were further subjected to preprocessing steps, including sorting, removal of duplicated reads, local realignment around small indels, and recalibration of base quality scores using SAMtools version 1.6, and Genome Analysis Toolkit (GATK) version 4.2.5. To make high-confidence predictions of mutation calls, we used MuTect2. The 1000 Genomes, gnomAD, and dbSNP datasets were used as reference databases for the known polymorphic sites. A variant-effect predictor was used to annotate each variant. Mutations with a minimum depth ≥ 20 and variant allele frequency of ≥2 were used in this study.

Targeted sequencing was performed to confirm the presence of *KRAS*, *NRAS* mutations in codons 12, 13, 59, 61, 117, and 146, and the *BRAF* V600E mutation. Microsatellite instability (MSI) status was analyzed according to the criteria provided by TSO500, which classifies CRC tissues into microsatellite stable (MSS), MSI-low (MSI-L), and MSI-high (MSI-H) groups, with MSS and MSI-L merged into one group. The TMB per megabase (MB) was calculated using the TSO500 panel. First, all *POLE* mutations were evaluated regardless of their pathogenicity. Patients with hypermutations were defined as having pathogenic *POLE* mutations, whereas those without hypermutations were classified as having non-pathogenic *POLE* mutations [12].

### 2.3. Data Presentation

Due to the limited number of cases with pathogenic *POLE* mutations, no statistical comparison was performed. Data from patients of foreign origin were not used, in order to properly assess the association between *POLE* mutations and clinicopathological characteristics in an ethnically Korean population. Clinicopathological characteristics and molecular profiles are presented descriptively using case counts and percentages. Continuous variables such as age and TMB are reported as individual values in the results and tables. In addition, histologic slides from patients with pathogenic *POLE* mutations were re-evaluated.

## 3. Results

### 3.1. Selection of Patients with Pathogenic POLE Mutation

**Figure 1:**
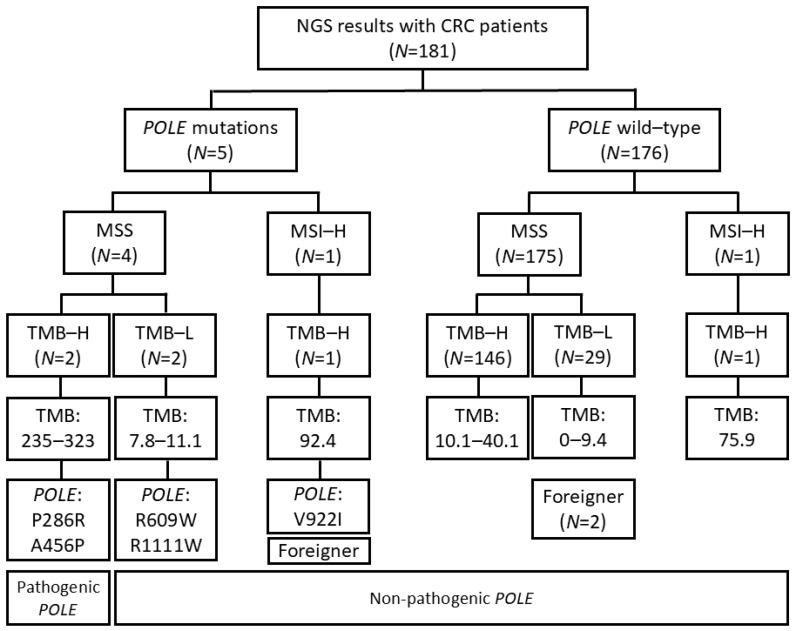
STROBE diagram of the colorectal cancer patient cohort. Patients were classified based on microsatellite status, *KRAS* mutation, *NRAS* mutation, *BRAF* mutation, and tumor mutation burden, including *POLE* mutation. H: high, L: low, MSI-H: microsatellite instability-high, MSS: microsatellite stable, TMB: tumor mutation burden.

The flowchart of patient selection for this study is shown in Figure 1. A total of 181 patients were identified with available NGS records, and *POLE* mutations were identified in five of them. Among these five patients, two had relatively low TMB values (7.8 and 11.1) and were therefore classified into the non-pathogenic *POLE* mutation group. One patient had a high TMB of 92.4 but was found to be MSI-H and was thus placed in the non-pathogenic *POLE* mutation group. All but one patient without *POLE* mutations were MSS. Among these patients, those with TMB < 10 mutations/MB were classified into the TMB-low (TMB-L) group (29 patients) and those with higher TMB into the TMB-high (TMB-H) group (146 patients). Three patients of foreign origin were excluded from the statistical analysis, although one of them had non-pathological *POLE* mutations and was included in the clinical description of patients with *POLE* mutations.

### 3.2. Association Between Pathogenic POLE Mutation and Clinicopathological Characteristics

The clinicopathological characteristics of the patients with CRC enrolled in this study are outlined in Table 1. The mean age was lower in patients with pathogenic *POLE* mutations than in those without (49.0 versus 61.2). All patients with pathogenic mutations were male, with one mutation in the right colon and the other in the rectum. Pathological studies showed that the former had a moderately differentiated histology and stage II disease, whereas the latter had a poorly differentiated histology and stage IV disease. No *KRAS*, *NRAS*, *BRAF* mutations or microsatellite instability were found in these two patients, and they showed a very high mean TMB of 279.2 mutations/MB.

### 3.3. Clinicopathological Features of Five Patients with POLE Mutations

The clinicopathological characteristics of the five patients with both pathogenic and non-pathogenic *POLE* mutations are described in Table 2. Two patients had pathogenic *POLE* mutations with a TMB > 200 mutations/MB. One patient (aged 48 years, male) had stage IV rectal cancer with an A456P mutation, poorly differentiated histology, and peritoneal metastasis. He underwent neoadjuvant chemotherapy followed by Hartmann’s operation. Subsequently, he was treated with second-line FOLFIRI (5-fluorouracil, leucovorin, and irinotecan) and third-line trifluridine/tipiracil plus bevacizumab; however, the disease continued to progress. Ultimately, he received immunotherapy once under terminal conditions, but died shortly after treatment administration, precluding assessment of its effects. Another patient (aged 50 years, male) had stage II ascending colon cancer, with a P286R mutation and moderately differentiated histology. He underwent right hemicolectomy followed by adjuvant chemotherapy and has remained disease-free from the initial surgery up to the last follow-up. Three patients, two female and one male, had non-pathogenic *POLE* mutations with a TMB of less than 100 mutations/MB. Two patients (all aged 62 years, one female and one male) had stage IV sigmoid colon cancers, with R111W and R609W mutations, respectively, and moderately differentiated histology. The female patient is currently undergoing sixth-line chemotherapy, although the disease continues to progress. The male patient is receiving second-line chemotherapy and has achieved a partial response. The remaining patient (aged 52 years, female) had stage II ascending colon cancer with a V922I mutation, medullary carcinoma, and MSI-H status, resulting in a TMB of 92.4 mutations/MB. She underwent right hemicolectomy followed by adjuvant chemotherapy and has maintained a disease-free status. All three patients had *KRAS* mutations and survived until the last follow-up. The overall survival (OS) of all 5 patients ranged from 9 to 44 months.

### 3.4. Histologic Features of Pathogenic POLE Mutation in CRC

**Figure 2:**
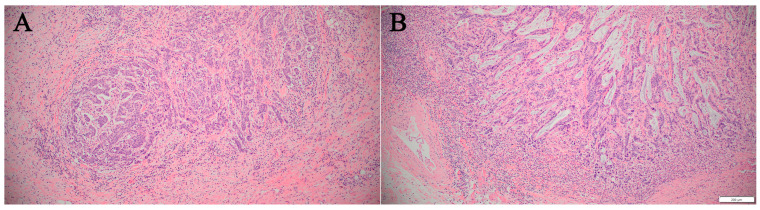
Histological features of *POLE*-mutated colorectal adenocarcinoma. (**A**) Predominantly poorly differentiated carcinoma with a solid, nested growth pattern (×100). (**B**) Conventional colorectal adenocarcinoma showing tubule formation with focal mucin production (×100).

We reviewed slides from two patients with pathogenic *POLE* mutations. In one case, although some tubular structures were observed, the majority of tumor sections displayed a solid nested growth pattern (Figure 2A). The other case was a conventional adenocarcinoma with tubular formation and partial mucin production (Figure 2B). Both patients had tumors that invaded pericolorectal soft tissues; however, no lymph node metastases were observed.

## 4. Discussion

Using NGS results from 181 patients with CRC, we compared the clinicopathological characteristics of patients with *POLE* mutations. Among the five *POLE* mutation cases identified, three (R1111W, R609W, and V922I) also had *KRAS* mutations, and one was MSI-H with a high TMB. However, because the remaining two patients showed relatively low TMB levels (7.8 and 11.1, respectively), they were considered to have non-pathogenic *POLE* mutations. In contrast, the two pathogenic *POLE* mutation cases had no *KRAS*, *NRAS*, or *BRAF* mutations and were classified as MSS. Both displayed ultra-hypermutations, with TMB values exceeding 200. One case was Stage II and the other Stage IV. Histologically, one case was an adenocarcinoma of moderate differentiation, and the other was poorly differentiated. Although immunotherapy was applied to the Stage IV patient with pathogenic mutations, early death precluded the assessment of treatment effects.

In 2016, a multicenter study was conducted involving approximately 6500 colorectal cancer patients to identify those with somatic *POLE* mutations and to perform clinicopathological analysis [9]. This study included 6517 patients who underwent *POLE* gene analysis, 66 (1.1%) of whom were found to have *POLE* mutations. The cohort with *POLE* mutations was compared with 833 dMMR (13.5%) and 5378 MMR-proficient (pMMR, 85.7%) patients. In the group with *POLE* mutations, patients were younger at diagnosis, predominantly male, mutually exclusive with dMMR status, more likely to present with relatively low-stage disease, and had higher histologic grades. These tumors were more frequently located in the right colon. Two groups showed statistically significant differences in all of these factors. Furthermore, among the 31 patients with *KRAS* mutation results, 30 harbored the wild-type allele.

Although our study had a smaller total sample size and fewer patients with *POLE* mutations, which limited the statistical significance of our findings, our results were consistent with those of this large-scale study. Similarly, Kawai et al. analyzed *POLE* mutations in 1052 colorectal cancer patients with CRC in 2021 [13]. This study focused on a Japanese colorectal cancer cohort, and *POLE* mutations were identified in 4 out of 1052 patients, indicating a slightly lower frequency. However, consistent with other findings, *POLE*-mutated colorectal cancers were significantly associated with younger age, right-sided colon location, poor histological grade, early stage, and *KRAS/NRAS/BRAF* wild-type status. In addition, Ahn and Ansari reported on *POLE* mutations in Korean CRC in 2016 [14]. In their study, *POLE* mutations were identified in 6 out of 83 patients (7.2%) with MSS early-onset CRC, while none were detected among 27 patients with MSS late-onset CRC. Consistent with other studies, *POLE* mutations occurred more frequently in the right colon [14].

There exists a series of studies on the use of ICIs in cancer patients with *POLE* mutations. A recent study compared the treatment results of 26 patients with CRC with *POLE/POLD1* proofreading-deficient mutations who received anti-PD-L1 and/or anti-CTLA-4 agents, with 516 patients from a cohort of patients with dMMR/MSI-H CRC who received the same treatment [8]. The clinicopathological features of the patients with *POLE/POLD1* mutations aligned with those described in the previous section. The overall response rate (ORR) for the mutation group was 89%, which was much higher than the ORR of 54% in the comparison cohort, with most patients showing a response within 6 months from the start of treatment. The 2-year progression-free survival (PFS) of the former group was also significantly higher than that of the latter (88% versus 60%). Another study in 2022 retrieved the data of 458 patients with cancer with *POLE* mutations from NGS reports of 14,229 patients with various cancer types, with 15% of *POLE* mutations identified as pathogenic [7]. Analyzing 82 patients receiving anti-PD-1/PD-L1 and/or anti-CTLA-4 regimens without other therapies, it was found that those with pathogenic *POLE* mutations had a higher rate of clinical benefit compared to those with benign mutations (82.4% versus 30.0%), as well as having a significantly superior median PFS and OS (15.1 months versus 2.2 months for PFS, 29.5 months versus 6.8 months for OS). These results indicate the potential role of pathogenic *POLE* mutations as indicators of positive responses to immune checkpoint inhibitors, considering the association of *POLE* mutations with higher TMB and stronger antitumor activity mediated by T lymphocyte infiltration [2].

The Cancer Genome Atlas (TCGA) established a molecular classification scheme to identify four subtypes of endometrial cancer (EC) based on genomic, transcriptomic, and proteomic features, one of which was a *POLE*-mutated subtype [3]. EC cases were assigned to this group based on the presence of established pathogenic mutations identified by gene sequencing, with exonuclease domain mutations in P286R, V411L, S297F, S459F, and A456P designated as ‘hotspot mutations’ [15]. *POLE*-mutated ECs have an exceptionally high rate of somatic mutations that frequently exceed 100 mutations/Mb, a relatively high proportion of C>A and T>G substitutions, and are mostly devoid of somatic copy number alterations or microsatellite instability [15,16]. Clinically, these cases occur in relatively young women, often with a higher tumor grade and endometrioid histological type, and have a highly favorable prognosis with a very low rate of recurrence or mortality [17,18,19]. These features of the *POLE*-mutated EC subtype are in accordance with our results in CRC, despite the small number of patients presenting with pathological *POLE* mutations, tending towards younger age, exceptionally high rate of TMB, little to no presence of MSI, and poor histological grades [8,9].

This study has several limitations. The analysis was limited to 181 patients with CRC with available NGS records from a single institution, possibly introducing selection bias in the choice of cohort. The small size of the total cohort and the rare presence of *POLE* mutations may have hindered the proper assessment of its prevalence in patients with CRC. Nevertheless, to the best of our knowledge, this is one of the few studies to provide detailed clinicopathological insights into *POLE*-mutated CRC in Korean patients. Immunotherapy was administered to a single patient; however, the terminal status and early death of the patient precluded the assessment of its effects. Despite the generally favorable prognosis of *POLE* mutation, the clinical course observed in this case provides valuable clinicopathological insights. Further studies on *POLE* mutations in CRC should incorporate a larger cohort from multiple clinical centers to determine its prevalence in clinical settings, confirm its propensity for early age occurrence and poorer histological grade, and assess its response to immunotherapy regimens.

## 5. Conclusions

In this study, we analyzed NGS data from 181 colorectal cancer patients and identified five cases with *POLE* mutations, two of which were pathogenic based on ultra-hypermutation and molecular features. Two patients had MSS and were wild-type for *KRAS*, *NRAS*, and *BRAF*. Notably, pathogenic *POLE*-mutated tumors showed an exceptionally high TMB (>200 mutations/MB), distinguishing them from MSI-H CRC. Although rare (about 1% in our Korean cohort), these tumors can be effectively identified through comprehensive NGS with TMB profiling or targeted *POLE* analysis. Our findings contribute valuable clinicopathological insights into *POLE*-mutated CRC and underscore the importance of broad molecular testing to guide immunotherapy decisions.

## Figures and Tables

**Table 1:** Comparison of clinicopathological characteristics between colorectal cancer patients with or without *POLE* mutations.

Characteristics		Pathologenic *POLE* Mutation (*n* = 2) (%)	Non-Pathogenic *POLE* Mutation (*n* = 179) (%)
Age	year old (mean, SD *)	49.0 (1.4)	61.1 (9.6)
Sex	Male	2 (100)	111 (62.0)
	Female	0 (0)	68 (38.0)
Tumor	Right	1 (50)	35 (19.6)
location	Left	0 (0)	69 (38.5)
	Rectum	1 (50)	75 (41.9)
Histologic	Well	0 (0)	9 (5.0)
type	Moderately	1 (50)	145 (81.0)
	Poorly	1 (50)	14 (7.8)
	Others	0 (0)	11 (6.1)
8th AJCC *	I	0 (0)	7 (3.9)
stage	II	1 (50)	18 (10.1)
	III	0 (0)	53 (29.6)
	IV	1 (50)	101 (56.4)
RAS/RAF mutational status	*KRAS* mutant	0 (0)	101 (56.4)
	*KRAS* wild type	2 (100)	78 (43.6)
	*NRAS* mutant	0 (0)	4 (2.2)
	*NRAS* wild type	2 (100)	175 (97.8)
	*BRAF* mutant	0 (0)	6 (3.4)
	*BRAF* wild type	2 (100)	173 (96.6)
Microsatellite status	MSS *	2 (100)	177 (98.9)
	MSI-H *	0 (0)	2 (1.1)
TMB *	mutation/MB * (mean, SD *)	279.2 (62.0)	8.1 (9.0)

* AJCC, American Joint Committee on Cancer; MB, megabase; MSI-H, microsatellite instability-high; MSS, microsatellite stable; SD, standard deviation; TMB, tumor mutation burden.

**Table 2:** Detailed clinicopathological information of five colorectal cancer patients with both pathogenic and non-pathogenic *POLE* mutations.

No.	Sex	Age	Tumor Location	Hist *	AJCC Stage *	*POLE*	*POLE* Significance	*KRAS*	*NRAS*	*BRAF*	MSI *	TMB * (mut/Mb *)	Tx *	OS *	Alive/ Death
1	M	48	Rectum	Poorly *	4	A456P	Path *	Wild	Wild	Wild	MSS *	235.3	Sx *, Ctx *, ICI *	16 mo *	Death
2	M	50	Ascending	Mod *	2	P286R	Path	Wild	Wild	Wild	MSS	323	Sx, Ctx	44 mo	NED *
3	F	62	Sigmoid	Mod	4	R1111W	Non-path	Mut *	Wild	Wild	MSS	7.8	Ctx	22 mo	Alive (mets) *
4	M	62	Sigmoid	Mod	4	R609W	Non-path	Mut	Wild	Wild	MSS	11.1	Ctx	9 mo	Alive (mets)
5	F	52	Ascending	Medullary	2	V922I	Non-path	Mut	Wild	Wild	MSI-H *	92.4	Sx, Ctx	34 mo	NED

* AJCC stage, 8th edition of the American Joint Committee on Cancer staging system; Alive (mets), alive with metastasis; Ctx, chemotherapy; Hist, histology; ICI, immune checkpoint inhibitor; Mut, mutant; mut/Mb, mutations per megabase; mo, months; Mod, moderately differentiated; MSI, microsatellite instability; MSI-H, microsatellite instability-high; MSS, microsatellite stable; NED, no evidence of disease; OS, overall survival; Poorly, poorly differentiated; Sx, surgery; TMB, tumor mutation burden; Tx: treatment.

## Data Availability

The original contributions presented in this study are included in the article/Supplementary Material. Further inquiries can be directed to the corresponding author.

## Supplementary Materials

The following supporting information can be downloaded at: https://www.mdpi.com/article/10.3390/diagnostics15080972/s1, Table S1: Panel gene list.
